# The groove hypothesis—music and dance reimagined: A sensory motor synchronization study

**DOI:** 10.1371/journal.pone.0328719

**Published:** 2025-09-22

**Authors:** Hiro Fumi Akao

**Affiliations:** Physiology Unit, Meiji International University of Health Sciences, Qchan Shinryosho Clinic, Tokyo, Japan; Shahid Chamran University of Ahvaz, IRAN, ISLAMIC REPUBLIC OF

## Abstract

Perfect synchronization, although essential in ensembles, has not been objectively evaluated. Further, the closely associated backbeat feel has not been adequately explained in the literature. This study introduces the groove hypothesis as a novel strategy for achieving perfect synchronization in musical ensembles, particularly in tasks involving backbeat feel. In ensemble playing, musicians must begin their movements before hearing the sounds of others to achieve synchronization. Conversely, in professional music settings, it is believed that target times are shared with an error of ±5ms based on experience, and this is considered perfect synchronization. Furthermore, while the word “groove” is used in various ways, here, it introduces the perspective of those who play groove. By examining the performance of participants using electronic drums with a 65-ms response delay, I identified two distinct synchronization strategies, shedding new light on how musicians and dancers manage rhythmic coordination. Based on the experimental results, the participants (ten university students and four dancers) were divided into Group A, which included six participants who could synchronize, and Group B, which included eight participants whose synchronization was delayed. Their p-values indicated that Group A used different strategies for the two plays. While the metrical swing theory explains why we can match a rhythm, I propose the groove hypothesis as a synchronization strategy for musicians: achieving perfect synchronization facilitated by imagining walking or running. The hypothesis suggests that we are aware of the motion of the center of gravity and that this awareness can be linguistically represented as a quiet inner “singing” in the brain. This idea is a new approach to music and dance performances and is also a proposal for cognitive science that suggests the possibility of linguistic imagery being involved in the perception of musical rhythms and synchronized movement of the body.

## Introduction

This study uses sensory motor synchronization (SMS) research with external stimuli, including an anti-phase task (sounding at the midpoint of metronome), to examine whether the well-known method of backbeat feel training facilitates perfect synchronization. The results of the experiment provided evidence of a cognitive change as a successful outcome of the anti-phase task, demonstrating the effectiveness of the training. Two independent strategies for achieving perfect synchronization were identified, and one of them was proposed as the “Groove hypothesis.” This is a new approach to how musicians and dancers perform rhythmic synchronization, based on a perspective that re-rates the existing metrical swing theory to physical movement. This theory is a method for imagining the swing of the center of gravity that accompanies walking or running as an internal clock. Another strategy is a characteristic derived from the skipping motion. It comprises a new proposal for the negative mean asynchrony (NMA) that has been observed in many participants and the cause of which has not been identified in the tapping paradigm [1,2]. Considering the “groove” as one that psychologists explore as the inner world of listeners [3–9], adding an alternate perspective of the performer’s inner world may indicate the location of what they are searching for. Therefore, the perspective of music teachers evaluating and conducting synchronous training provides a new way to understand rhythm, movement, and cognitive processes with simultaneous linguistic imagery.

### Metronome training of the backbeat feel

**Fig 1:**
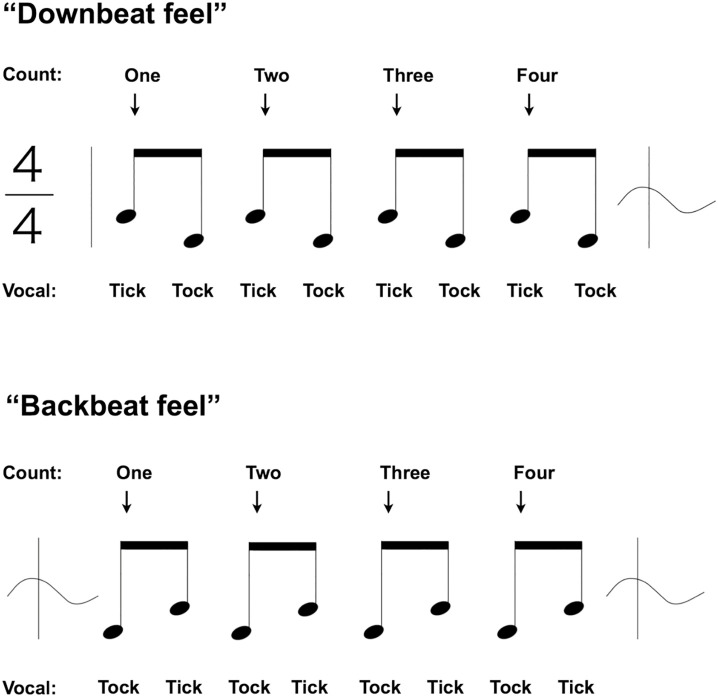
Downbeat Feel and Backbeat Feel. A 4/4 time signature means that within one bar, the denominator is the type of note (quarter-note), and the numerator is the number of that note (four), meaning that a metronome beat (tick) is counted as four in a bar. This is the downbeat feel, while the backbeat feel counts your own striking sound (tock) as a beat.

Every song has a beginning, an end, and a rhythm that is counted. The usual downbeat feel involves counting the drum sound at the midpoint between consecutive metronome clicks as “tick-tock,” while the backbeat feel counts it as “tock-tick” (Fig 1). Moreover, when aligned with the “bounce” that these two sounds have under the backbeat feel, their length is equal. An internal reference clock is necessary to synchronize this bounce with movement, which can be created by the up-and-down rhythmic movement of the lower limbs; musicians call this state “groove.” If one can imagine a gait in which one foot is synchronized to a metronome, the other foot becomes the reference for the strike. Counting the other foot is then equivalent to the backbeat feel. The backbeat feel involves rhythm training, wherein the metronome is used as a reference for a player who plays the backbeat by consciously altering the musical count; the first beat is played by the players themselves. Without the courage to take the first step, perfect synchronization cannot be achieved.

### Research on musical rhythm and humans

The common use of the metronome is for a downbeat, as used in studio recordings. Although the term “backbeat feel” is well known, its method and theory have not been explained in previous studies. The ability to synchronize with external stimuli has been researched as SMS, and various metronome effects have been reported (these studies have been reviewed twice [1,2] through in-phase (downbeat) and anti-phase (downbeat or backbeat) tasks. However, the extant literature fails to clarify the cause of NMA. Nonetheless, the relationship between musical rhythm and humans continues to be studied by music psychologists, who have defined NMA as the participants’ tendency to overestimate their own synchrony [4,5], conceptualized micro-timing as unexplained displacement, and described the keyword “groove” as a sensory scale for psychostatistical methods. In recent years, Duman et al. [10] have been working on a new definition of groove, taking up the point made by Frühauf et al. [3] that “a satisfactory definition of groove must include specific rhythmic structures and how these structures are performed,” and that made by Senn et al. [11] regarding the incompleteness of the current groove model. They have called for the development of a substantive measure of the groove [10].

Although top-down research is now prominent, an important variable in bottom-up research had already been discovered during the late 20th century: the swing ratio. The swing ratio is a parameter of music composition software (e.g., “Performer; 1985”, MOTU, Cambridge, MA, USA), which connects two and three beats in a linear ratio and makes it possible to comprehensively de-script basic rhythms in popular music. Subsequently, music rhythm teachers were able to establish a method by considering the characteristics of swing as human kinetic characteristics. To better understand the idea that the swing ratio is not related to the tempo, the reader can listen to the sequence (around 15-min long) that changes from swing 0–100%, made with two unaccented hi-hat notes, in the video “Metrical Structure of the Swing Rhythm” (https://youtu.be/OuMU0yzMfpI; created by the author).

### Major experimental designs

To eliminate NMA in synchronous measurement, music tests are sometimes conducted in preliminary [12], but it is assumed that using electronic drums with a 65-ms sound delay would suppress NMA. Furthermore, it is a major load on the target action.

The number of participants was estimated based on the assumption that a minimum of three successful participants (Group A) would be required to show meaningful numbers in the t-test of two performances. The results, fortunately, yielded six participants in Group A, which increased the reliability of the p-values. The small number of total participants can be justified by the fact that the number of general participants (regular music listeners), including university staff who requested to participate, is unlikely to increase the number of people in Group A due to the difficulty of the experiment. In a study of synchronized movement with music by Tranchant et al. [12] using 101 university students as participants, one participant was excluded from the analysis because their synchronization was too high. This experiment was designed to find participants with the same predisposition as the one excluded.

### Evaluation of perfect synchronization

The first evaluation of metronome training is synchronization: if participants can achieve a mean of ± 5 ms (perfect synchronization), which can be called micro-timing, under a 65-ms sound delay load, they can be considered professionals. The result showed that six participants were able to achieve perfect synchronization, who were assigned to Group A. Two performances were tested, and the p-values for Group A suggested that they used a different strategy to achieve successful synchronization. Therefore, the training effect is recognized as a result of the appropriate application of the bias (instructions) in the anti-phase task. However, the t-test does not reveal content.

### Evaluation methods for groove strategy

If swing in music theory is considered a variable, four independent groove strategies can be assumed as the participants’ internals, and the dependent results can be used to evaluate and instruct the synchronization methods proposed by the music teacher.

The four independent groove strategies are as follows: (1) quarter-note jumpers, which correspond to the first and second plays in Group B; (2) eighth-note jumpers, the first play in Group A; (3) eighth-note walkers or runners, who played the second play alternately with both hands in Group A; and (4) quarter-note skippers who played the second play with one hand in Group A. Backbeat feel can be applied only to (3), but perfect synchronization can also be technically achieved with (4). In fact, (4) can also be considered a cause of NMA.

However, there is a lack of factors in the estimation of the multivariate equation that prove this. Hence, the method is built on music theory and the trainer’s experience as an initial report. Thus, it is important to investigate individual thresholds for the onset of swing feeling. Empirically, non-musicians begin to feel the swing at a swing ratio of 50–60%, but musicians can play at 30% swing; the backbeat feel training is expected to improve that threshold.

### Instruction methods for perfect synchronization

The groove hypothesis is proposed as a methodology for (3) [eighth-note walkers (or runners)]. If one is aware of walking as bouncing images, they can be linguistically manipulated to synchronize. This suggests that we are aware of the trajectory of our center of gravity when we walk. As instruction for perfect synchronization, I describe how grooving in a walking rhythm is a rational way to teach. Further, for musicians who play groove with swing, an implicit shared pulse pattern is eighth-note swing created with walking.

In the main text, following the materials and methods, the results are described in detail, as this is to support the validity of the data; further, as in the case of the NMA interpretation, there is the possibility of pointing out individual instruction queries. Although the analysis of the electrocardiogram and EEG recordings failed, it was felt that EEG could be used as an internal measurement, and “swing” and “groove” are discussed.

The groove hypothesis is expected to open up new perspectives not only for new approaches to music and dance performances but also for understanding the cognitive processes that link rhythm and movement. Considering swing rhythm as a characteristic of physical movement represents the key to solving many related questions.

## Materials and methods

**Fig 2:**
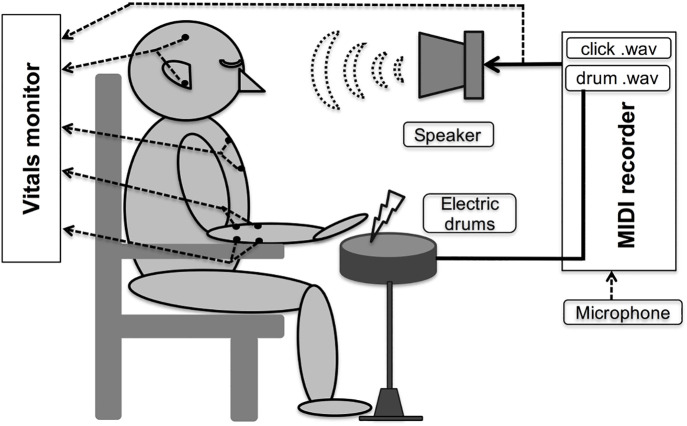
Devices. The participant repeatedly strikes the drums, aiming for the midpoint of the metronome and relying on the sound from the speaker. Further, electric drums are sounded with a delay of 65 ms. A metronome is generated by the MIDI system to record the timing and intensity of the strikes. Simultaneously, the vital recorder records (1KHz-16-bit) speaker sounds, Electroencephalography (EEG), Electrocardiogram (ECG), and Electromyogram (EMG).

The experimental task corresponds to the performance of the simplest ensemble tune by two percussionists. One player is a machine; the rhythmic skills of the other player, a human, will be evaluated. However, this player will use low-performing (65-ms delay) toy electronic drums. A rhythm player focusing solely on timing must: 1) follow the onset absolute time of the score written as a rule (downbeat feel in Fig 1); 2) be stable with little disturbance in strike time; 3) be continuous (5 minutes); and 4) follow the tempo of this task (1 Hz)—the maximum of 60 outcomes (mean = 58.3 ± 3.6) obtained per minute is enough to apply a mean value, which can be used to estimate the player’s target time. The standard deviation (SD) of the obtained mean value is zero for the machine player. For humans, a smaller absolute value of SD means less blurring. The maximum length of a typical popular music piece is approximately 5 minutes; thus, 5-minute means were evaluated (including continuity).

### Ethical approval

The experiments in this study were conducted after obtaining approval from the Research Ethics Committee of Meiji International University of Health Sciences, Kyoto (no. 26-55) for a master’s degree study, “Quantitative Research for Sensory-Motor Control” (2014–2016). A detailed explanation of the purpose and content of the study was provided to all participants, and they gave written informed consent with university staff in attendance. Four minors under the age of 20 also obtained written consent from their guardians.

### Participants

Fourteen individuals volunteered to participate; specifically, participants included 10 university students (five men and five women; average age: 20.8 ± 1.5 years) who were highly familiar with the music and listened to it daily, as well as four professional dancers (three men and one woman; average age: 23.8 ± 4.8 years). The absence of physical defects was confirmed verbally, but ventricular extrasystoles were detected in one case, although this was unknown to the participant. A drummer in the music club was asked to participate, but they declined. Participants were recruited using on-campus posters; the judo and music clubs, as well as the nursing department at the university where the experiment was set, also assisted in the recruitment process. The period for recruiting participants was from October 24, 2014, to December 8, 2014. The experiment was conducted from December 9, 2014, to July 24, 2015. The recruitment notifications made it clear that rhythm ability would be judged. The professional dancers were acquainted with the author. One teacher of hip-hop dance participated, but the data were not used as the device was misconfigured, leading to data contamination. The nature of the task made it important that participants be remarkably familiar with music, which informed the choice of participants. The author (a 56-year-old man and former music teacher) was also recorded under the same conditions as a reference equivalent to a music teacher (participant #0).

### Devices

Electronic drums were used as the input device, and musical composition software (Digital Performer 7; MOTU, Cambridge, MA, USA) was used to reproduce the metronome and drums, as well as record the strike timing and intensity. The metronome sound was a 14-ms-long rimshot with peaks at 500 Hz and 1.5 kHz, and the drum was a 40-ms-long small drum with sound peaks at 200 Hz and 500 Hz, and a low-cut filter (30 Hz/-60 dB) was applied. The sounds were monitored by a small speaker placed 1.2 m in front of the participant at shoulder height. At the same position as the participant’s head, the sound pressure of the metronome was 70 dB (316 Hz to 8 kHz), and room reverberation was 200 ms (RT60–60 dB); the sound pressure of the drum was 70–75 dB, and reverberation was 220 ms. In addition, there was a delay of 65 ms in the sound from the speakers of the electronic drums.

### Recorded data

Performance data were recorded as musical instrument digital interface (MIDI) data (the de facto standard for data transfer of electronic musical instruments, with a resolution of less than 1 ms for the timing and intensity of the strikes with a 65 ms correction. Moreover, the electroencephalography (EEG; C3-A1, C4-A2), electrocardiography (sternal guidance), electromyography (left and right flexor digitorum brevis and extensor carpi radialis shortus), and the original sound of the speakers (temporal reference) were recorded on a second personal computer using a body surface electrical activity recorder (Vital Recorder II and Bimutas II; Kissei Comtec, Matsumoto, Nagano, Japan; Fig 2). The free software R (v.3.2.1; 2015; R Foundation for Statistical Computing, Vienna, Austria) was used for analysis and plotting.

### Experimental procedure

For the in-phase task, participants were instructed to make a sound simultaneously with the metronome, but discrimination between the two tones required a time frame of 15–20 ms or longer between them [13,14]. In the anti-phase task, conversely, participants were instructed to make a sound at the midpoint of the metronome; at the experimental tempo of 1 Hz (beats per minute [BPM]: 60), there was a 500-ms interval between the two.

Participants received the following instructions before the experiment began: “The experimental task is to listen carefully to the sound of the speakers and play the drums at the midpoint of the metronome. Two 5-min sessions will be held. If the metronome sound is ‘cuts’ and the drum sound is ‘tong,’ you will hear ‘cuts-tong, cuts-tong.’ In the first play, you will strike to match this beat. When the performance stabilizes, you will realize that you can hear the metronome sound after your own drum sounds, such as ‘tong-cuts, tong-cuts.’ If possible, try this on your second play. The first time, you will strike with both hands alternately; however, in the second play, you may use only one hand instead of alternating.”

Subsequently, participants practiced for approximately 1‒3 min until they were satisfied with the device, and were then instructed to close their eyes and focus on the sound while wearing an eye mask; movement of body parts other than those of the hands at the end of the armrest were suppressed by a cushion. The performance was then recorded twice following the procedure in Table 1.

**Table 1:** Procedure.

Time	Details/“Narration”
**300 s**	Rest/“Rest for the first 5 min. After 5 min, the metronome and a perfect example will be played every 5 min”
**300 s**	Only click sounds are presented
**300 s**	Click sounds and accurate drum sounds are presented
**120 s**	Rest/ “After 2 min, the metronome will start. Strike with both hands alternately, aiming for sounds at the midpoint of the metronome click”
**300 s**	First play
**40 s**	Interval/ “This time, when your playing becomes more consistent, try to be aware of the clicks that follow your strikes. Additionally, you should play with one hand instead of alternating”
**300 s**	Second play
**180 s**	Rest
	Written questionnaires and interviews

## Results

The experiment was performed as per the procedures in Table 1, and the data from 28 performances (number of strikes = 245–300, mean = 291.4 ± 12.1) were obtained from 14 players for each 5-min task, with a 65 ms correction. For two recordings of one strike, the preceding record of lower intensity was deleted as a missed touch.

### Data normality

The histogram of deviation from the midpoint of the metronome sound with a width of 10-ms showed a Gaussian distribution for all performances. As an action that is aimed at a specific target time, the outcome can be assumed to be normally distributed.

### Correlation between mean and SD

**Fig 3:**
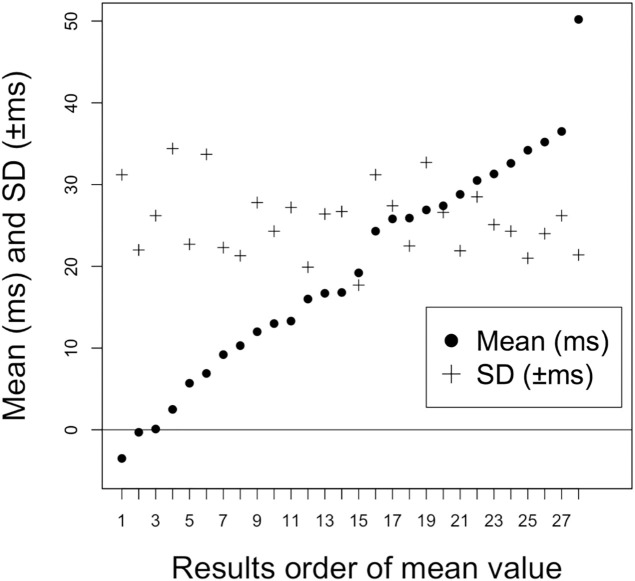
Mean Asynchrony and Standard Deviation for All Performances. The absolute values of the 5-min mean and its SD are plotted on the vertical axis, with each performance on the horizontal axis in order of the smallest value first. While the mean of the target time is widely distributed, many SDs are in the ± 20-ms range. The mean value is 19.6 ± 13.0 ms, and the mean absolute value of SD is 25.6 ± 4.2 ms. Pearson’s correlation coefficient test shows that the correlation coefficient is 0.24, a weak correlation implying that the values may be considered independent.

Performances were arranged on the horizontal axis in the order of 5-min mean asynchrony; the asynchrony and absolute value of the SD were plotted on the vertical axis. Positive mean asynchrony, manifesting as “delayed action,” was observed in many performances, and its value decreased (asynchrony approaching zero), representing an improvement in performance. The mean asynchrony for all 28 performances was 19.6 ± 13.0 ms, and the mean absolute value of the SD was 25.6 ± 4.2 ms. Pearson’s correlation coefficient was 0.24; this correlation is weak, but the values can be considered independent (Fig 3).

### Musical judgement and grouping

**Fig 4:**
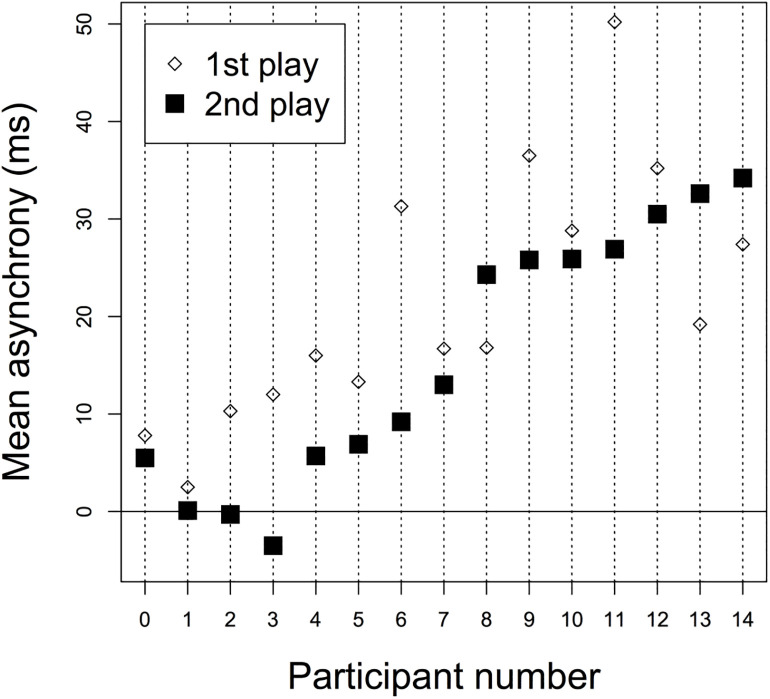
Participants’ Results. The two performances are plotted on the vertical axis with the mean value, which is close to zero, as the grade. On the horizontal axis, numbers are assigned to participants in order of the magnitude of the absolute value of the 5-min mean of the second play.

Participant numbers were assigned in the order of their second play; 5-min mean asynchrony values for first and second plays were plotted on the vertical axis. The mean asynchrony for the 1-min sections of each performance showed that six people achieved ± 5 ms (Table 2). These six people constituted Group A, which achieved perfect synchronization, while the other eight participants with delayed synchronization constituted Group B. Specifically, participants #1 to #6 were assigned to Group A and participants #7 and thereafter to Group B (Fig 4).

**Table 2:** Grouping Criteria.

Means of 1-min sections	#0	#1	#2	#3	#4	#5	#6	#7	#8
**± 5 ms**	5	3	4	3	2	2	1	0	0
**± < 5–10 ms**	3	3	3	1	2	2	2	1	1

This table shows the number of targets achieved in 1-min sections; ± 5 ms achievers were included in Group A.

### t-tests

A two-tailed paired t-test of the means of the first and second asynchronies yielded p = .01 for Group A and p = .78 for Group B. In the first play, both groups showed asynchrony positive values, with a mean of 14.2 ms for Group A and 30.7 ms for Group B. However, in the second play, Group A significantly improved its performance by 3 ms, whereas Group B’s performance, at 26.7 ms, did not improve significantly. Thus, Group A might have used a different strategy in the second play, and the results confirmed the effectiveness of the training (Table 3). However, this does not prove that the backbeat feel was applied; this detail is discussed musically in the “evaluation with groove strategies” section. Additionally, in Group B, three participants showed increased asynchrony for the second play (see below). However, significant p-values were obtained for only three people, indicating that there is high normality of the data. This test becomes more reliable as the number increases (Table 3).

**Table 3:** t-Test Results for Groups A and B.

Group	No. of participants	1st play	2nd play	*p*
**Group A**	6	14.2 ms	3.0 ms	.011
**Group B**	8	30.7 ms	26.7 ms	.779
**Decreases in B**	5	33.5 ms	24.4 ms	.721
**Increases in B**	3	21.2 ms	30.4 ms	.048

### SD

SD is an index of target time stability in the tapping paradigm [2], which is reported at ± 16 ms for a professional percussionist (the same tempo as in this experiment [15]). The minimum value of the 5-min mean was recorded in Group B by #13 (a professional dancer) at ± 17.7 ms in the first play. In Group A, #4 (a piano player) recorded ± 13.1 ms in the 3rd-min section of the first play as the minimum value for the 1-min sections. The other values were in the 20-ms range, with a slight increase in means from ± 25.9 ms to ± 26.3 ms for Group A and from ± 23.8 ms to ± 26.5 ms for Group B for the two performances.

Large disturbances were observed in three cases. Participant #1 (a professional tap dancer) made a key adjustment to move the target time approximately 30 ms earlier in the 4-min section of the first play, yielding a considerable increase in SD. In the interview, she explained that she produced a downbeat feel with a feeling of delay for the first 3 min and then attempted a backbeat feel. The result showed a mean of 13.3 ± 26.0 ms for the first 3 min and a 5 min mean of 2.5 ± 34.4 ms. In the second play of #5 (non-performer), SD was greatly disturbed only in the 4-min section for unknown reasons; perfect synchronization (1.1 ms) was achieved in this section. In the second play of #11 (piano player), SD increased significantly only in the 3-min section. This may have occurred after ventricular extrasystoles—which were observed multiple times at rest—appeared simultaneously with the strike. Aside from the case of large intentional adjustments for #1, the target time for each participant was presumably maintained even when stability was disturbed. Although there were no group differences in SD, individual differences were observed.

Playing 65-ms response-delayed drums is unusual, even for a musician, and sounds tend to be delayed in musicians’ percussion. Musicians adjust to their own criteria by operating more slowly if they think their strike sound is too fast or too slow. If so, the range of adjustment is the SD, and the mean value serves as its own standard (internal clock) for the individual. Thus, a constant SD for the participant during the transition from the lagging position toward the target would imply a change in their internal clock.

### Bilateral differences

All participants performed with both hands alternately in the first play; the mean asynchrony of all participants for the first play was 18.6 ± 25.4 ms and 20.7 ± 24.1 ms for the right and left hands, respectively. Although the dominant hand tended to exhibit stronger performance, #13 had the opposite result. No correlation was observed between hand performance and stability (smaller SD), although left–right differences could be observed between individuals. Individual details of the performances are shown in Table 4.

**Table 4:** Comparison of Performances.

Participants	Mean asynchrony		(1)	(2)	(3)	(4)	(5)		(6)
	First play	Second play	L–R difference	SD	Strength dif.	SD	Second strength		Dominant hand in the second performance
#0	7.8 ms	5.5 ms	≃	L < R −15.4%	R > L + 137%	L < R −27.7%	△	113.8%	Alternating
#1	2.5 ms	0.1 ms	≃	≃	≃	L < R −19.0%	△	119.6%	Alternating
#2	10.3 ms	−0.3 ms	R < L −4.0 ms	≃	≃	R < L −11.1%	▼	81.8%	Mostly right
#3	12.0 ms	−3.5 ms	≃	≃	L > R + 143%	R < L −45.0%	▼	51.2%	Completely right
#4	16.0 ms	5.7 ms	R < L −6.6 ms	≃	L > R + 119%	L < R −16.4%	△	127.6%	Alternating
#5	13.3 ms	6.9 ms	≃	L < R −13.7%	L > R + 121%	R < L −12.8%	–	95.8%	Random
*#6	31.3 ms	9.2 ms	L < R −19.3 ms	≃	≃	≃	–	93.4%	Alternating
#7	16.7 ms	13.0 ms	R < L −3.3 ms	≃	R > L + 117%	R < L −43.5%	▼	85.7%	Alternating
#8	16.8 ms	24.3 ms	R < L −5.7 ms	R < L −26.8%	L > R + 114%	R < L −27.9%	▼	65.9%	Random
#9	36.5 ms	25.8 ms	R < L −7.8 ms	R < L −12.0%	≃	≃	–	102.7%	Alternating
#10	28.8 ms	25.9 ms	≃	≃	≃	≃	△	131.6%	Alternating
#11	50.2 ms	26.9 ms	≃	≃	L > R + 133%	R < L −10.2%	–	96.1%	Random
#12	35.2 ms	30.5 ms	≃	≃	R > L + 127%	L < R −12.2%	–	99.2%	Mostly right
*#13	19.2 ms	32.6 ms	R < L −3.2 ms	L < R −15.7%	R > L + 137%	L < R −35.6%	△	120.5%	Random
#14	27.4 ms	34.2 ms	≃	R < L −12.1%	R > L + 134%	L < R −13.0%	△	156%	Completely right

The first play was performed alternately by all participants. (1) Target time difference between hands (better than 3 ms). (2) Higher stability of the target time (smaller SD, 10% or less). (3) Higher strength (110% or more). (4) Higher strength stability (smaller SD, 10% or less). (5) Average intensity of the second play compared with the first (△10% or more, ▼ −10% or less). (6) Performance of the second play. *Participants #6 and #13 used a pen and chopsticks, respectively, with their left hands.

### Strength differences

No correlation was observed in the difference in strength between left and right hands in the first play and SD; some players were more stable on their stronger side, and some on their weaker side. No correlation was observed between left- and right-hand strength, their SDs, left and right asynchronies, or the SDs in the previous section. In addition, five participants performed the second play more strongly than the first, and four performed it weakly; however, no group difference was observed (Table 4).

### Double-action technique

Electromyography showed that muscle activities that did not reach striking (so-called blank hitting and double-action, mainly with the right hand) were observed among two participants each in Groups A and B: for #1 in the 4th- and 5th-min sections of the first play and throughout the second play, for #10 throughout the first and second plays, and for #2 and #13 throughout the second play. No group differences were observed.

### NMA

In the laboratory metronome paradigm, NMA occurs unconsciously among many individuals, corresponding to the target time; however, it is smaller among musicians [1,2]. In this experiment, negative means were found only in the top six performers and positive means in the other eight. The 65-ms sound delay on the electronic drums was enough load to counteract the NMA; however, top performers could aim for the target time, suggesting many tended to consciously create a delay in synchronization practice.

A negative mean for the 1-min sections of the second play was found for #2 and #3 and lasted for more than 3 min; both played the second play mainly with the right hand. For electronic piano, keyboard speed is converted into pressure, which is practical for the player. In this case, striking hard is the same as striking fast; however, the second play’s striking strength for these two participants was reduced to 82% for #2 and 51% for #3 (Table 4).

Striking weakly on the electronic drum is like tapping with fingers. As a technique, strong striking is an open-loop movement and cannot be adjusted after the start of the movement, while weak striking is a closed-loop movement and can be adjusted [16,17]. One-handed play is considered musically in the “Groove” section below*.*

### Increase in asynchrony

Mean asynchrony increased after two plays for three participants in Group B; their two performances were significant, p = 0.048 (Table 3). In a post-performance interview, #13, a professional dancer (animation dance/robot dance), responded, “I did not do as instructed because I got bored the second time.” The second play’s strength was set to 120%; #8 (a black belt judoka) learned to play the piano during his childhood and had 66% strength in this play. Similarly, #14 (a black belt judoka), who had no playing experience, had strikes with 156% strength in the second play. However, for the 1-min sections of the first play, #8 had 5.5 ± 30.8 ms, #13 had 10.9 ± 14.2 ms, and #14 had 12.5 ± 26.6 ms, which is close to Group A’s results.

### Electrocardiography

Electrocardiography measurement was useful to ensure the safety of the participants. In this experiment, ventricular extrasystoles were monitored with participant disturbance. In autonomic function research, the balance of the autonomic function has been estimated by extracting the power spectral density from heart rate variability time-series data: the two-periodic structure of low frequencies (LF) and high frequencies (HF). LF (0.04–0.15 Hz) and HF (0.15–0.40 Hz) power spectra were extracted from the sternal potential by Fast Fourier Transform approximately every minute, and sympathetic nervous system activity was examined on the basis of the LF/HF ratio. Specifically, HF (approximately 0.25 Hz) is an indicator of parasympathetic activity known as sinus arrhythmia; LF (approximately 0.1 Hz) is the Mayer Wave, a baroreceptor reflex. Thus, LF/HF is an indicator of sympathetic activity [18]. However, this method could not be adopted because several cases increased LF/HF immediately after the performance when sympathetic activity should have been inhibited. This was attributed to deep breathing, but this index could be applied only in a resting state. By contrast, performance in the experiment was not attempted in a resting state as it involved considerable exercise and mental tension for some individuals.

### EEG

The EEG recordings were conducted as a primary investigation of the relationship between the feeling of elation or exaltation during groove. In recent years, scholars have reported the relationship between finger tapping tasks using audiovisual beat stimulation and EEG [19], and the relationship between drumbeats, pupil measurement, and EEG [20], but the present study explores the brain activity behind the responses to visual stimuli. Due to the limitation of the equipment used, only two measurement points (left and right) were used, and only the frequency of the individual subject was examined. Although many studies have investigated the relationship between EEG and auditory stimuli, this experiment involved spontaneous movements, and it was not clear whether the recording itself would be possible. In a preliminary experiment, artifacts were detected with elbow extension but not with slap and tap, and alpha-band activity was visible during the performance; therefore, it was added to the measurements.

During the performance, most of the center frequency of the background EEG was recorded as the alpha-band in all participants except one (#10), whose alpha-band was unmeasurable. Center frequencies showed a large bimodal distribution; however, statistical verification was impossible because the theta‒alpha region boundary was unclear, and consciousness was not controlled during non-performance. Although sleep was not evaluated, the anti-phase task is deemed more difficult than the in-phase one [1,2]. Further, resisting a 65-ms delay in sound is a physical movement that requires mental concentration; thus, it is difficult to perform when drowsy.

Although statistical validation failed, it was shown that background EEG can be recorded during performance. Also, the cushions used to suppress artifacts in the recording ensured that no body movements were made by participants.

### Participants’ attributes

Two participants had no experience playing musical instruments, four were professional dancers, two were amateur electric bass players, four were amateur piano players, and four belonged to an amateur band (Table 5). Further, #5, a black belt judoka, had no experience playing musical instruments and did not dance (only the dancers danced regularly), but his performance met the benchmark for Group A.

**Table 5:** Participants’ Backgrounds.

Musical background	Electric bass players	0, 2, 3
Piano players	**4**, 7, 8, 11
Drum players	11, 12
Vocalists	9
Dancers	**0**, **1**, **6**, 10, 13
No experience playing a musical instrument		**5**, 14
Black belt judoka		**0**, **4**, **5**, 8, 14
(Group A is marked in bold)		

The questionnaire (administered after the experiment) revealed that all participants listened to music daily, and except for #11 (a trained taiko player), all had played the arcade game “Taiko no Tatsujin” (“Taiko: Drum Master,” Bandai Namco Entertainment, Tokyo, Japan), which is very popular in Japan. The fact that all participants had drum-playing experience may have contributed to the large number of synchronous successes (Group A), as “Taiko no Tatsujin” has the same component—strike time evaluation—as this experiment. With the exception of #9, an amateur band vocalist, all participants said that they could silently play music in their heads. Regarding difficulty, only #7 answered that the task was easy and she could perform it well: all her 1-min sections were stable (10-ms level) throughout the two performances, suggesting that she was a well-trained player; however, she belonged to Group B (Table 6).

**Table 6:** Questionnaire After the Experiment.

“How difficult was the task?”	
It was difficult.	**0**, **4**, **5**, 12
I don’t think it was difficult.	**1**, **3**, **6**, 8, 9, 10, 11, 13, 14
It was easy.	**2**, 7
**“How were your results?”**	
I could do it well.	7
I could do it somewhat well.	**0**, **1**, **2**, **3**, **4**, **5**, **6**, 8, 9
I don’t know if I did it well.	10, 12, 13, 14
I couldn’t do it well.	11
(Group A is marked in bold)	

## Discussion

First, the metrical structure of the swing rhythm, as the basis for all further arguments, will be explained. Second, the theoretical explanation of backbeat feel to obtain perfect synchronization will be established, and the various conventions assumed in the field of rhythm training will be discussed with regard to swing. The discussion continues with the notion of groove, which could be the indispensable internal clock to perform a backbeat feel. The researcher assumes that jumps, walks, and runs are basic movements of groove as actual body movements and infers the groove strategy of the participants based on the relation to the tempo of performing these movements. This establishes a method of instruction. The next step is to propose a groove hypothesis that explains how musicians perform synchronization.

### Swing

Musical “swing” rhythm appears to be a mysteriously handed down technique with no adequate academic explanation; hence, a dictionary expression must be quoted. The Merriam-Webster Dictionary defines it as a “steady pulsing rhythm,” “steady vigorous movement characterizing an activity or creative work,” and “jazz dancing in moderate tempo with a lilting syncopation.” Historically, swing became popular in the United States from the mid-1930s to the mid-1940s in big-band dance music. As a basic rule, musical rhythms are broadly divided into two and three beats; however, in the late 20th century, music composition software (e.g., “Performer; 1985”, MOTU, Cambridge, MA, USA) was equipped with a parameter called “swing ratio.” This quantitatively showed that swing is a qualitative rhythm between two and three beats.

### Temporal structure of swing

**Fig 5:**
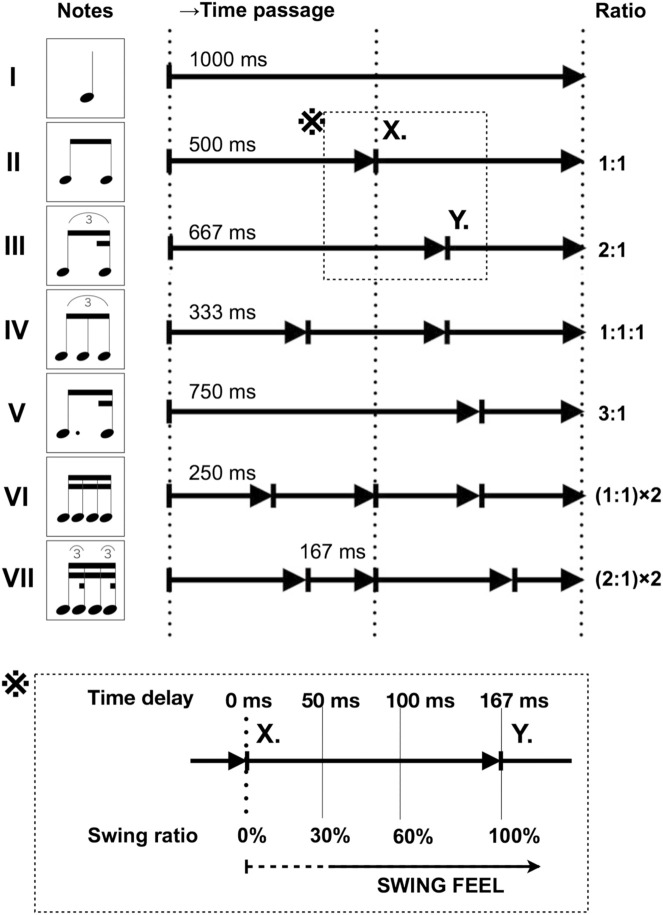
Basic Rhythm Patterns (BPM: 60). BPM represents the tempo of a song, expressed by the number of quarter-notes per minute. The figure shows the temporal resolution of quarter-notes as basic rhythm patterns. I: is the quarter-note divided into two eighth-notes; II: in jazz, the middle note of the triplet is often extracted and played as III; III: this is called the quarter-note swing, the basic form of swing; IV: this is a triplet, which divides a quarter-note into three equal parts; V: this is a sixteenth-note feel that matches the first and fourth notes of **VI.** Going from III to V is sometimes called a hard swing; VI: this is a straight sixteen-note feel; VII: the sixteenth-note feel can be a swung eighth-note, as in **VII.** ^*^If the second note of II, named X, goes late to Y, the swing feel of III begins to occur.

The repetition of two notes (duration 1:1) is a basic two-pattern. When the second note is delayed, the swing feel begins to occur; then, 2:1 becomes a swing matched as a perfect triplet (three beats). This excludes the second note of the triplet, yielding swing (https://youtu.be/OuMU0yzMfpI; created by the author). Fig 5 shows the time division of the basic pattern at 60 BPM.

In Fig 5(I), if the metronome is applied to a quarter-note, the note length is 1,000-ms. The task is to produce sound at the point where it is bisected. The metronome and the struck sound are notated as two eighth-notes that bisect a quarter-note (Fig 1 and Fig 5(II)).

In jazz, rhythm is often played (Fig 5(III)), except for the middle note of the triplet, and this is the basic form of swing. The rhythm based on Fig 5(II) is a straight (flat) “eighth-note feel,” but as the second note is delayed, swing begins to occur in Fig 5(III). The description of Fig 5(III) is called a “quarter-note swing” because the swing occurs within a quarter-note. The description of Fig 5(IV) is a triplet of quarter-notes divided into three equal parts.

If Fig 5(III) is set to a 100% swing ratio and Fig 5(II) is set to a 0% swing ratio (this may be Weber’s ratio; however, as a de facto standard, musical software uses percentiles for practical purposes), the swing feel starts to appear at approximately 30%; at approximately 50–60%, even without knowledge of musical rhythm, most people will feel a difference in the rhythm between 5(II) and a straight rhythm. Moreover, a 150% swing is the “sixteenth-note feel” of Fig 5(V); however, up to approximately 120%, it produces a swing feeling similar to that at 100%. The difference between 80% and 100% is also difficult to distinguish. In addition, Fig 5(VI) is a straight sixteenth-note notation. However, as in Fig 5(VIII), an “eighth-note swing” can be applied; “half-note swing,” which is a half-note divided into two, is also a playing style. In a 4/4 time signature, a half-note swing is demonstrated by the audience clapping hands on the second and fourth beats. This does not cause disruption, even if performed very late. Rhythm players keep the promised tempo and swing ratio, while the soloist can use various swings as rhythmic ornamentation, which also can be pronounced slightly before the onset timing of the regulated backing band, corresponding to the lilting syncopation in the dictionary meaning. The fact that the two and three beats are connected by a linear ratio merits the term “swing theory.”

### Qualitative nature of the swing ratio

Friberg and Sundström [21] noted that swing works by delaying the onset of the even-numbered subdivisions of each beat. Moreover, Frane and Shams [20,22] described its properties, supporting time division by the swing ratio, which they expressed in numerical ratios. However, in this study, the swing ratio is expressed as the percentile of sensory utility. Additionally, Frane and Shams’s [22] eighth- and sixteenth-note swings are described as quarter- and eighth-note swings, respectively. Câmara [23] and Frane [24] analyzed funk and hip-hop recordings and reported that drummers often use an eighth-note swing from 0 to 30%.

Marchand and Peeters [25] algorithmically estimated the swing ratio of the quarter-note and observed different trends across musical genres, showing that even the quarter-note swing is different from classical music. The conductor’s intention may change the tempo and swing ratio in a song. In jazz, the swing ratio varies and may be used as ornamentation. However, popular music has a constant tempo and swing ratio, which are independent parameters. The swing ratio is constant regardless of tempo unless physical limits are exceeded (e.g., in composition software), indicating that rhythm is a ratio as well as pitch. This means that even if there is a physical limit, such as the inability to distinguish between straight and swing as the tempo increases, popular music has a constant tempo and a swing ratio that are independent of each other. Marchand and Peeters’ [25] report of lower swing detection in reggae and zero detection in hip-hop makes sense if it is an eighth-note swing performance.

The swing ratio is suspected to be less common because, in the 1990s, when karaoke used only a small amount of MIDI data in a low-speed telecommunications environment, swing ratio settings were indispensable for manipulators to create precision copies of the original tune. However, the broadband environment of the 2000s eliminated this technical work [26]. Since its discovery, the swing ratio value has sometimes been added to the score; however, in an ensemble, it is often set subjectively by the bandmaster, the drummer, or collectively by those who know the original music. The ratio can also be accurately—and perhaps more easily—conveyed by playing a short tune. In this experiment, the ratio was also instructed in short phrases of the language chunks. Further, the process of recording short phrases and repeating them as chunks is often used in song-writing. In this context, it is not necessary to know the numeric ratios. Currently, the most difficult part of this process is matching the onset of the playback sound sources owing to the rising edge before the first peak.

### Specific method of the backbeat feel

**Fig 6:**
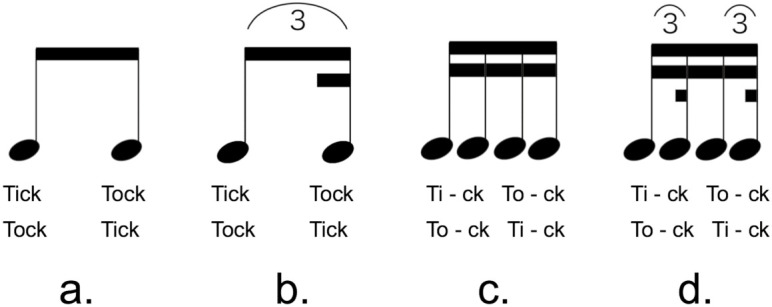
Backbeat Feel with Swing. **a.** Metronome clicks (tick) and drum sounds (tock) can be noted as two eighth-notes of equal duration. **b.** If the second note is delayed, a quarter-note swing occurs as the ratio of the two note lengths. **c.** Two notes can be divided into sixteenth-notes. **d.** An eighth-note swing can be applied to each word, and by aligning the ratios, the onset position of the two words can also be aligned.

First, one must be aware of the sounds, replacing them with language (e.g., the onomatopoeia of “tick-tock”). The specific method of the backbeat feel is explained in Fig 6: “tick” and “tock” are notated in eighth-notes, as in (a). If the eighth-note is divided and the sixteenth-note is not considered, it may result in a quarter-note swing, as in Fig 6(b); however, as two-mora (-beat) words, they can also be notated in detail in sixteenth-notes, as in Fig 6(c). In this straight sixteenth-note feel, notes are evenly spaced and thus hard to compare unless pitch and timbre are distinct. However, Fig 6(c) can be swung by an eighth-note, as in Fig 6(d). By swinging each of these two words and consciously saying them in a different order, we can adjust them to match. In music theory, as a phrase pattern, the two notes in Fig 6(a) coincide with the first and third notes of Fig 6(d), giving the absolute eighth-note position.

### Delayed synchrony and swing ratio

Synchronization movement delay is expressed by the swing ratio. The first play mean of 30.7 ms for Group B was approximately 18% swing, whereas that of Group A was approximately half or 9% swing. The Group A baseline of 5 ms corresponds with the 3% swing. Moreover, the first play of #11, which had the largest delay among all performances (Fig 3), was stable at 50.2 ± 21.4 ms. This participant is an experienced piano player who prefers traditional Japanese music and is also a *tsuzusumi* (traditional drum) player. Therefore, this participant performed at a stable 30% swing.

Many plays were often delayed, and swings occurred, although the experimental task should not be swung because of the metronome. If a swing feeling does not occur, the rhythm may sound almost correct; thus, a positive mean asynchrony can be considered an unconscious swing.

### Delay tolerance

In Group A, there were 10 ms or more of delayed synchronizations in the first play. This raises the question of whether a difference of approximately 10 ms is acceptable as synchronization. The SD, which is the deviation from the target time, is ± 16 ms (at 1 Hz) for professional drummers [15]. If the SD is approximately ± 15 ms from the target time, it may be considered the same as the target time. If the discrimination of two tones requires more than 15‒20 ms [13,14], this can be considered a detection of delay, and a delay of half the discrimination time (i.e., approximately 10 ms) may be acceptable as synchronous. For example, if a drum sound has a duration of 20 ms, a delay of approximately 10 ms in the matching sound may be acceptable as an ornament.

Additionally, for drum sound tones at 100 Hz, we must listen to one frequency (10 ms) to identify the pitch; after this, vibrations can be felt. Digital conversion to electronic devices always causes delay; if the delay is more than 20 ms in an electronic drum, the high-frequency hi-hat will feel the delay, although the low-frequency bass drum will be acceptable because vibrations are always felt after the event.

### Delay due to distance

Sight can be blocked out by closing one’s eyes, but hearing and somatosensory perception are retained. Repp [1] noted that research on SMS has focused mainly on the auditory modality, which contradicts most other perception–action research conducted in the visual domain, typically considered “where the action is” in sensory perception psychology. This discussion stems from the perspective of performance instruction that can be performed without visual information.

In an ensemble, players are at a physical distance from each other, and to synchronize, some must play slightly earlier. The diffusion of sound takes approximately 15 ms for 5 m and approximately 30 ms for 10 m. It also takes time for the brain to transmit commands to the muscles and for the muscles to respond by contracting. This means that players in an ensemble must move before they hear the sounds of others. Fortunately, musical performance is completely predictable when based on a written score, the metronome does not betray expectations, and other tools are used to facilitate synchrony, such as orchestra conductors and marching bands’ physical marching, in which all participants are essentially rhythm players.

According to a strict music teacher, a “delay” appears if a play is tailored to others, and rhythm players are taught that they must take the first step themselves. If a delay of less than approximately 15-ms is perceived as a coincidence, a radius of 5 m is comfortable for those playing to feel others’ presence.

### Synchronization conventions

Synchronization is required in ensembles, and sounding “too fast” is noticeable to all within a given onset time rule. In addition, there are deviations that players are not aware of; however, as the target time is normally distributed, the risk of being noticeable can be reduced if the median is shifted to the right (delayed). A slight delay may be more acceptable than producing sound too early. This may lead to the musical habit of delaying a little and following others.

In music notations, a song has a beginning and an end, and rhythm is counted according to the time signature. In the downbeat feel, the pattern includes a delay corresponding to the asynchrony, which may be acquired by the individual, much like a linguistic “dialect.” The degree of the accent can be expressed as a swing ratio, and a backbeat feel can be used to align the accent. In music performance, the tendency to tailor to others and thus be late seems common.

### Groove

Groove means nothing without the definition of swing because the physical expression of swing is groove. In relation to music and according to the Merriam-Webster Dictionary, *groove* is defined as “an enjoyable or exciting experience” and “a pronounced enjoyable rhythm.” Malone [5] defined two distinct concepts of groove: “the feel of the music” and the psychological feeling (induced by music) of wanting to move one’s body. This study adds the musicians’ perspective to the definition of groove, considering it as another concept: in an ensemble, each player needs an internal clock, and groove is the first candidate. Groove can be created as a lower body movement pattern and used as a vehicle for performance by the upper limbs. To groove is translated as “to ride” in Japanese [*noru*; noun form *nori* “(a) groove”]. Once one experiences groove as a physical movement and then practices it, one should be able to imagine the movement internally.

### Basic motions of groove

Some musicians play without motion, some sway their heads back and forth (or up and down or left and right), some mumble, some tap one foot, some bounce up and down, some walk, and so on. Essentially, people groove in different ways; however, what is common is a constant repetitive motion. Regarding human movements, we are familiar with jumping, walking, and running as repetitive movements that defy gravity and move us upward. Walking becomes running when the tempo increases, and running is considered an alternating jumping motion. Musicians in the field use “bounce” with groove. Bouncing movements may be accomplished with the lower limbs.

### Tempos of walking and running

**Fig 7:**
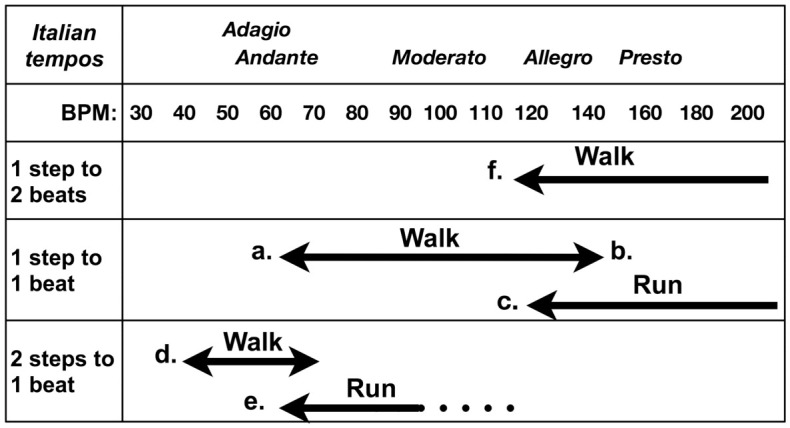
Walking and Running for Metronome Tempos. This figure subjectively shows the tempo at which a person can walk and run smoothly to the metronome beats. Walking turns into running as the tempo increases. **a.** The tempo of 60 BPM is one step per beat according to the metronome. This allows smooth walking but is quite slow. In the Italian tempo description, this corresponds to *Adagio*—gently—or *Andante*—at a walking pace (actually, slightly slower). At the slower 50 BPM, it is impossible to walk smoothly as a series of movements. **b.** The tempo of 120 BPM is quite fast; however, it is a speed at which one can walk lightly (*Allegro*—lightly) up to 140 BPM. **c.** “Running” can also be jogging, starting at approximately 120 BPM. **d.** The lower limit of smooth steps per beat is 60 BPM; however, it is possible to walk at a slower tempo with two steps per beat. Nevertheless, if 60 BPM is one beat and one can take one step, one cannot be smooth at 30 BPM without taking two steps. In general, the slower tempo is usually in triplet time; however, the lower limit is approximately 40 BPM, and 30 BPM is not often observed. As musical notation was originally written to make it easier for performers to play and not write something that cannot be played, there is a limit to how slowly it can be matched. **e.** Although running is possible at 120 BPM, it is possible to run at 60 BPM if one takes two steps per beat. **f.** While 120 BPM is a tempo that can be walked at two steps per beat, the clicking sound between steps is too straight to groove to. This suggests a swing in every step.

Musicians groove to maintain rhythm and perform by grooving to the rhythm. Under gravity, when one adjusts to metronome beats, body movements cause take-offs and landings; one likely uses landings as a reference point, akin to heel strikes in walking, marching, or dancing. The player must be aware of the point of landing; however, the take-off will have been completed before that point (the solutions are discussed in the “Motion of groove and its image” section). In any case, the basic movements resulting in stable repetitions are walking and running, and smoothly repeated jumps can be accomplished at the same tempo as running (see the note to Fig 7 for details).

### Jumping

At rock concerts, the audience often jumps to fast-tempo songs, and keeping in time with the beat can be difficult unless the tempo is somewhat fast. The tempo for a jump that can be repeated smoothly is like that for running, and one step per beat would be smooth at 120 BPM or higher. At a slower tempo, the time period without a jumping motion is longer, and the motion is not continuous. However, if the jump is made twice per beat, it is as though the tempo has been doubled, resulting in a smooth movement. To attempt a backbeat feel from a swing pattern, resetting to step (or jump) again is necessary. This can also be done with a skip, as described below.

### Walking

For performers who must quickly find and groove to a tempo and swing ratio, walking is recommended to maintain rhythm. The walk, the most familiar method of human movement, is stable, sustainable, and versatile, and it lets the player take steps with confidence. A smooth walk at one beat per step is approximately 60‒140 BPM (see the note to Fig 7 for details). Consider the image of continuous walking: one need not reset the backbeat feel but only change the count to see which side is first; then, the eighth-note swing can be imagined with each step, and when the left and right swings are aligned, upright bipedal walking is complete.

### Skipping

Skipping is jumping with both feet slightly offset at the landing. According to developmental kinesiology, the adult gait begins after the acquisition of this skill; however, some people cannot skip well or can skip only in one direction. This gait is also a basic movement in martial arts, such as *waza* in judo, as well as in sideways-riding sports, such as surfing. These have been explained as examples of the emergence of primitive reflexes [17].

In the first play of the experiment, all participants were forced to use both hands alternately; however, in the second play, they were free to use whichever hand they preferred. Those who performed the second play mainly with one hand may have been predisposed to skip movements in addition to being dominant (Table 4). In Group A, #2 and #3 struck almost exclusively with the right hand in the second play and had noticeable negative means. In addition, #5 (a black belt judoka) achieved 1.1 ms in the 4th-min section of the second play; however, during this time, he used only his right hand, and at other sections of the second play, he switched sides approximately every 10 s. In the 5th-min section, he achieved a negative mean of −5.5 ms. He could skip either left or right, and it is possible that he performed the first play with alternating skips. Incidentally, in the black belt examination, all *kata* techniques must be done on both sides [27]. In any case, these three participants had already achieved ± 5-ms mean in the first play, which they performed by alternating both hands.

Some people prefer to skip instead of walking on some occasions. A skip is a vector added to a jump with the same up-and-down rhythm as a jump. It is a unilateral movement, and one cannot alternate both hands to strike while skipping, although the offset landing rhythm becomes a swing. However, as a repetitive action, it is the same as jumping; thus, at slower tempos, repeated resets would be used.

Additionally, in the experiment, there was a clear swing between the somatic sensation of the strikes and the speaker sounds 65 ms later. This could also be a bias, but it was not considered. There was no claim of drum sound lateness from participants.

### Skipping and NMA

In this experiment, continuous negative asynchrony means were observed in one-handed players. As in many martial arts, pushing in one direction allows for fast movements; if one has a predisposition for skipping movements and lacks an awareness of time division (non-musicians), using only one hand to play may be related to the appearance of NMAs in synchronization experiments. Moreover, this technique can be used to create swing, an important part of jazz, referring to the sensible syncopation used in the dictionary description of swing, which is different from syncopation on the score, represented by notes. In a standard drum set, the first beat is struck with the right hand and foot, just like the skipper.

### Evaluation with groove strategies

As groove strategies, participants attempted to manage the task using the following methods. It can also be described in musical notation. Fig 6(a) is assignment, Fig 6(b) is quarter-note jumpers or skippers, Fig 6(c) is eighth-note jumpers, and Fig 6(d) is eighth-note walkers or runners.

#### Quarter-note jumpers.

Jumping to the metronome beat appears to be the most common method; it is assumed that Group B (except #7) did this. Jumping must be at least twice the task tempo to be smooth. Consequently, their strikes had delays of 25–30 ms (5-min mean), using a backbeat feel, which should require an understanding of sixteenth-notes; however, owing to the large delays, this may have been impossible.

During the second play, #8, #13, and #14 (Table 4) decreased asynchrony to approximately 10 ms in the first play but could not decrease it further. This is because they tried to imagine an eighth-note jump from a quarter-note jump; however, the swing that arose made continuing difficult, and they returned to a quarter-note jump for the second play. Participant #13 said that he did not follow the instructions in the second play. This participant is an animated dancer, and #10 is his dance partner. These two participants usually performed a relatively robotic dance with few smooth movements, which created a delay. To attempt a backbeat feel from a swing pattern, one must reset to begin the step (or jump) again. Because of their typical movements, these two participants were thus consistently resetting their own actions, reducing the smoothness of their actions.

#### Eighth-note jumpers.

Group A performed an eighth-note jump for the first play. Their tempo was doubled to 120 BPM and was smooth (in Group B, #7 may have had this in both plays). However, there was an asynchrony of approximately 15 ms, which is half of that of Group B. This tempo allows for walking or running two steps per beat; hence, decreasing asynchrony at this time seems to be a change from jumping to walking or running. Backbeat feel can thus be applied for smoother walking or running. See Fig 3 for all result plots; the lack of results at approximately 20 ms may show the distinction between quarter-note and eighth-note jumpers.

#### Eighth-note walkers or runners.

A frequency of two steps per beat at this tempo allows for smooth walking or running and an awareness of the sixteenth-note. When applying the backbeat feel, the side that starts the step count must simply be swapped; no step resetting is necessary. In Group A, #1, #4, and #6 alternated strikes in the second play and might have followed this strategy and succeeded at the task.

#### Quarter-note skippers.

In Group A, #2, #3, and #5 performed the second play with one hand and were considered to prefer the skipping motion. This allowed them to jump earlier, delaying the metronome position and creating a quarter-note swing. Further, one can adjust to the exact eighth-note position by delaying the position of strikes. Such an advanced technique may be characteristic of swing in jazz. Those in Group B who performed with one hand may have skipped the on-beat in the quarter-note; however, their results were the same as those of the quarter-note jumpers. Another possibility is that of backbeat walkers who land between metronome beats. If a backbeat walk tempo can apply to many music tempos, it should be more comfortable than running. However, it is uncomfortable to walk when the metronome sounds like a straight eighth-note in one step; moreover, it is difficult to imagine sixteenth-notes without training.

### Instructions

Experimental results showed that there were no group differences in many individual factors. It can be said that Group B did not know or just could not find the method. Group B first trained with jumps from a slower to a faster tempo, then attempted to jump twice per beat (eighth-note action) and then walked to it. In addition, in the first play, participants were asked to alternate the use of their hands, which would not interfere with their walking but would inhibit riding sideways. Using the eighth-note walk as background and the backbeat feel for linguistic awareness, the swing ratio of the two notes was aligned; both sides of the walk were equal. Therefore, this is also the basic training of dance.

Repp [1] summarized two error-correction processes in SMS: one, “primarily under cognitive control, possibly operating by adjusting the internal timekeeper’s cycle,” is equivalent to grooving; the other is “mainly automatic and works by phase resetting.” If asynchrony is eliminated by a backbeat feel, to get a backbeat feel, one must create a groove to stabilize period control and linguistically control the phrase. Further, one should “chant” a phrase in practice. Obviously, at a fast tempo, physical movement is limited, while precision is difficult at a slower tempo. A phrase that cannot be chanted well at this time cannot be played well. Therefore, singing is also a good way to train rhythm.

The experimental task required a ruthless straight eighth-note feel that should not be swung; however, those who did find a swing in the eighth-note (or technically in the quarter-note) could succeed in the task.

### Motion of groove and its image

As a continuous movement pattern for creating groove, jumping, walking, and running have similar vertical movements of the center-of-gravity in the sagittal plane. An extension of the knee joint and flexion of the ankle joint (both concentric contractions) produce upward acceleration, and these eccentric contractions produce deceleration on landing. If jump and gait cycles are kept constant and take off strongly, dwell time is extended, resulting in a swing in the time ratio to the deceleration motion. Groove is a state in which the center-of-gravity is swinging at a fixed ratio: that is, the center-of-gravity holding stability mechanism is in operation.

As for the actual movement in walking, the heel–ground contact has a considerable impact, and although it can be used as a reference, similar to a dance step, it disturbs the sine wave. The upward part of the wave will draw a parabola owing to acceleration. If the deceleration movement draws a negative parabola, the sine wave will be in order. In this manner, gait without heels will greatly increase the degree of freedom of vertical movement adjustment. Therefore, the toe contact with the ground can be recognized as the timing of the take-off, not the landing. Subsequently, a strong bounce in a continuous motion will result in a swing of the center of gravity. Furthermore, walking adds a swing in the frontal plane.

Since we are aware of this movement, we are able to imagine it. Todd and Lee [28] posited four propositions for rhythm perception: (i) as a form of vestibular perception, (ii) as causing external and internal guidance for somatic topic expression, (iii) as links from the limbic system to internal guidance pathways mediating “dance habits,” and (iv) as innate vestibular reward mechanisms.

Patel [29] and Patel and Iversen [30] proposed the vocal learning hypothesis, suggesting that only certain species can synchronize with musical rhythms. Indeed, a parrot that can vocalize is a master of groove [31]. For humans, body movements (including vocalizations) can be naturally synchronized with temporal music, while the movement itself may cause a feeling of grooving. We can groove with verbal imagery that does not involve movement. We can sing internally.

### Groove hypothesis

Based on the proposition that the swing ratio has a qualitative nature, I propose the “groove hypothesis.” This idea explains how players play groove from the perspective of teachers.

In popular music performances, rhythm players must be able to imagine a groove in tune with others to maintain rhythmic stability. The pulse pattern shared by the musicians is the quarter- or eighth-note swing, equivalent to a walking or running step as a repeated bounce. The degree of bounce of each step is quantitatively set as the swing ratio, and the increase in the swing ratio provides a groove feel. The step can also vibrate to produce beats. This internal clock of walking and running can be imagined as a center-of-gravity stabilizing mechanism, and its up-and-down swing can be manipulated with linguistic imagery as singing a swing quietly in their head. The performers then perform on this swinging vehicle.

### Prospects for the clinical use of swing and groove

Although the nature of EEG’s alpha-band activity remains unclear, and the relationships with hearing and vision have been investigated, there are few reports on relationships with movement. In this study, the central frequency of background EEG during the performance was recorded as the alpha-band in most participants. Although statistical verification was impossible, it remains true that the exercise in this experiment did not inhibit the appearance of alpha-band activity.

Elation or exaltation in the groove may be a type of trance. Scholars have reported that alpha-band activity in the EEG of dancers in a trance state increased in the Kecak dance in Bali [32–34]. They believe that alpha-band EEG activity correlates with monoamine secretion, thus indirectly representing reward system activity. Therefore, this could be a variable in the inner quest.

Repp and Su [2] noted a lack of research evidence but anticipated the clinical application of these findings. Swing is important for gait rehabilitation. If the voluntary groove activates the reward system by releasing monoamines in the brain, we can expect that the regulation of mental functions without medication and alpha-band activity of the EEG may be used as indicators of it. Therefore, this suggests that a malfunction in the regulation of physical movement can have psychological consequences. Meanwhile, the dictionary defines groove as “enjoyable experience.” It may be simply achieved by walking with your heels up.

## Conclusions

We should be aware of the trajectory of the center of gravity during walking as a sensation. Especially for the rhythm players, to take the first step independently, the metronome backbeat feel is considered effective for perfect synchronization, as theoretical understanding and practice are based on it. The count swapping in the backbeat feel (i.e., the quiet singing of simple pulse patterns to imagine the groove of physical motion) and its manipulation are possible, as linguistic images support the groove hypothesis that musicians are implicitly synchronized with the swing at all times.

There are certainly fewer than six successful participants to prove the hypotheses put forward. Among them, it is certain that those who were able to synchronize perfectly even under a 65ms load were detected as Group A of participant predisposition. Professional tap dancers (participant #1) can also be considered music players, although professional music players are still not measured. Researching professionals may be useful for exploring unknown variables.

Given the variety of extant research, this study did not verify previous findings; instead, it focused on the evaluation of rhythm training and its description. I hope that the fundamental exploration of the synchronization of music and movement will begin once again. However, the qualitative nature of swing must first be confirmed. Conversely, eighth-note swing may be detectable from the participants’ onomatopoeia. Regarding NMA, it would be prudent to examine participants’ predisposition toward side-riding; for groove, it would be prudent to investigate the relationship between swing rhythm, monoamines, and EEG, although this requires verification under different gravity conditions. However, as music is performed by human movement, the swing rhythm should be an expression of the kinematic character of the body. Neuroscience must consider these characteristics.

## Supporting information

S1 Data All Data. All CSV files contained in the “All data” folder constitute the entirety of the data.
(ZIP)
